# Distinct brain activation patterns linked to clinical symptoms during hand grasping in Parkinson's disease

**DOI:** 10.3389/fnagi.2026.1895333

**Published:** 2026-09-21

**Authors:** Kaili Wang, Jing Guo, Dan Liu, Qiong Wu, Jin Qiao

**Affiliations:** Department of Rehabilitation Medicine, The First Affiliated Hospital of Xi'an Jiaotong University, Xi'an, Shaanxi, China

**Keywords:** brain functional activation, constipation, freezing of gait, functional near-infrared spectroscopy, hand grasping task, Parkinson's disease

## Abstract

**Objective:**

This study aimed to investigate the characteristics of brain functional activation during a hand grasping task in patients with Parkinson's Disease (PD) using functional near-infrared spectroscopy (fNIRS), and to further explore the clinical factors associated with the level of regional brain activation.

**Methods:**

Fifty-nine PD patients were enrolled. Demographic data (age, sex, education level) and clinical characteristics (disease duration, Hoehn-Yahr stage, UPDRS-III score, side of onset, motor complications, cognitive function, activities of daily living, non-motor symptoms, and levodopa equivalent daily dose) were collected for all participants. Oxygenated hemoglobin (HbO_2_) signals from multiple brain regions were acquired using fNIRS during a hand grasping task. With sex, age, and disease duration as covariates, multiple linear regression analysis was used to examine the relationships between clinical variables—including side of onset, constipation, REM sleep behavior disorder (RBD), Freezing of Gait (FOG), UPDRS-III, Montreal Cognitive Assessment (MoCA), and Hoehn-Yahr stage—and brain activation metrics (both temporal-domain and advanced features).

**Results:**

Among the 59 PD patients, 38 (64.4%) had constipation and 17 (28.8%) had freezing of gait (FOG). Under the left-hand task, constipation was significantly positively correlated with parameters in 13 brain regions (pFDR < 0.05), mainly including the bilateral dorsolateral prefrontal cortex, left frontal eye field, and left premotor area (regression coefficients: 0.0033–0.8538). No other clinical variables showed significant associations. Under the right-hand task, FOG was significantly negatively correlated with the full width at half maximum (FWHM) of the right primary motor cortex (β = −8.52, 95% CI: −12.98 to −4.06, pFDR = 0.010). No other clinical variables showed significant associations; however, larger studies are needed to confirm these findings and to evaluate potential subtle effects, particularly for RBD.

**Conclusion:**

In PD patients, constipation is associated with cortical hyperactivation in the prefrontal-premotor areas during the non-dominant hand task. Conversely, FOG correlates with reduced hemodynamic persistence and shortened activation duration in the right primary motor cortex (M1) during the dominant hand task. fNIRS can act as an objective tool to explore the cortical mechanisms of motor and non-motor symptoms in PD, providing hypothesis-generating evidence for potential targets that warrant further investigation in future repetitive transcranial magnetic stimulation (rTMS) intervention trials.

## 1 Introduction

Parkinson's disease (PD) is a common neurodegenerative disorder of the central nervous system, with its prevalence increasing with age. In China, the prevalence of PD among individuals aged over 60 years is approximately 1.37%, corresponding to more than 3.6 million patients (Qi et al., 2021). By 2030, the number of PD patients in China is projected to account for about 50% of the global PD population, reaching approximately 4.94 million cases (Dorsey et al., 2007). Clinically, PD is characterized by motor symptoms (e.g., bradykinesia, rigidity) and non-motor symptoms (e.g., autonomic dysfunction, sleep disturbances), which together severely impair patients' quality of life (Kalia and Lang, 2015; Postuma et al., 2015). Current therapeutic interventions for PD have notable limitations. Long-term levodopa therapy leads to progressive loss of efficacy in nearly all patients, and more than half develop dyskinesia (Kim et al., 2020). Although deep brain stimulation (DBS) improves tremor and rigidity, the incidence of gait or balance deterioration—particularly freezing of gait and falls—is significantly higher after DBS than after medication alone (Janssen Daalen et al., 2025), suggesting that DBS offers limited benefit for axial and non-motor symptoms. Repetitive transcranial magnetic stimulation (rTMS), a non-invasive neuromodulation technique, has shown promise for alleviating PD symptoms. However, the lack of standardized and individualized cortical target selection criteria currently results in heterogeneous therapeutic outcomes (Chen et al., 2025; Rektorova et al., 2025).

Consequently, identifying precise cortical targets for rTMS is of substantial clinical importance. Nevertheless, current evidence is constrained by the use of single stimulation targets and substantial heterogeneity across patient cohorts. As a result, the cortical functional modulation profiles associated with different clinical phenotypes of PD remain inadequately characterized (Rektorova et al., 2025)—a critical bottleneck limiting individualized target selection. Therefore, elucidating the relationships between multidimensional clinical indices and cortical modulation patterns during motor tasks in PD patients is an essential prerequisite for advancing individualized rTMS interventions. This goal aligns with the broader trend in health research toward objective assessment and personalized intervention, in which machine learning approaches have proven valuable for health risk identification and stratification (Du et al., 2025, 2026b). Functional Near-Infrared Spectroscopy (fNIRS) is a non-invasive neuroimaging technique that indirectly reflects neural activity by measuring concentration changes in oxygenated and deoxygenated hemoglobin as near-infrared light passes through brain tissue (Arenth et al., 2007). Compared with functional Magnetic Resonance Imaging (fMRI), fNIRS is more robust against motion artifacts and thus better suited for investigating motor tasks (Wu, 2020). Moreover, fNIRS signals show high consistency with blood oxygenation level-dependent (BOLD) signals (Sasai et al., 2012), and the technique has been successfully applied to evaluate brain function during walking, finger movement, and other tasks in PD patients (Kashou et al., 2016; Gramigna et al., 2017; Klempir et al., 2019; Guevara et al., 2024). Hand grasping is a typical gross hand movement primarily involving large upper-limb muscle groups. This action relies on coordinated regulation across multiple brain regions, including the sensorimotor cortex and prefrontal cortex, and can sensitively reflect the central functional status of motor planning, execution, and sensory integration in PD patients. Thus, hand grasping serves as an ideal experimental paradigm for investigating motor-related brain functional abnormalities in PD. The recently proposed Somato-Cognitive Action Network (SCAN) offers a broader framework for interpreting these cortical activation patterns within an integrated motor-cognitive perspective. The SCAN is a large-scale brain network that interleaves with classical effector-specific motor regions in the primary motor cortex (Gordon et al., 2023) and integrates motor control with cognitive, autonomic, and bodily signals. In PD, SCAN dysfunction—particularly hyperconnectivity with subcortical structures—has been implicated in both motor and non-motor symptoms, and effective neuromodulation may act by normalizing this network (Ren et al., 2026). The SCAN thus provides a useful framework for interpreting phenotype-specific cortical activation patterns in PD. Based on the above background, this study employed fNIRS combined with a hand grasping task paradigm to systematically characterize cortical activation patterns and their associations with various clinical variables in PD patients. Specifically, this study aimed to: (1) evaluate the independent associations between these clinical variables—including motor symptom severity, side of onset, cognitive level, levodopa equivalent daily dose, constipation, REM sleep behavior disorder (RBD), and freezing of gait (FOG)—and cortical activation parameters; and (2) identify phenotype-specific activation patterns to provide objective neuroimaging evidence for clinical subtyping and to generate testable hypotheses for individualized rTMS targeting.

## 2 Materials and methods

### 2.1 Participants

This cross-sectional study consecutively enrolled 59 patients with idiopathic Parkinson's Disease (PD) who were treated at the Department of rehabilitation medicine, the First Affiliated Hospital of Xi'an Jiaotong University (Shaanxi Province, China), between January 2022 and February 2026.

Inclusion criteria were: (1) age ≥18 years; (2) diagnosis of idiopathic PD according to the Movement Disorder Society (MDS) revised diagnostic criteria for Parkinson's disease (2015 version) (Postuma et al., 2015).

Exclusion criteria were as follows: (1) left-handedness; (2) previous intracranial surgery (including stereotactic lesioning and deep brain stimulation); (3) upper limb dysfunction due to prior trauma, deformity, or other causes; (4) history of other neurological or psychiatric disorders (e.g., subdural hematoma, stroke, encephalitis, epilepsy, schizophrenia, major depressive disorder); (5) bilateral motor symptom onset at disease initiation; (6) uncertain side of onset due to patient recall failure or other reasons; (7) critical illness or severe cardiac, hepatic, or renal dysfunction based on medical records.

This study was approved by the Ethics Committee of the First Affiliated Hospital of Xi'an Jiaotong University (Approval No.:XJTU1AF2023LSK-202). All participants provided written informed consent prior to enrollment.

### 2.2 Methods

#### 2.2.1 General data collection

General data were collected for all PD patients, including sex, age, education level, onset form, disease duration, medical history, family history, history of psychiatric disorders, and medication status. Medication status included the types, doses, and duration of current medications. All medication doses were converted to levodopa equivalent daily doses (LEDD), including levodopa-LEDD, dopamine agonist-LEDD (DA-LEDD), and total LEDD. The conversion formulas were based on reference (Tomlinson et al., 2010) as follows:

Levodopa-LEDD = levodopa standard-release tablet × 1 + levodopa controlled-release tablet × 0.75 (+33% if co-administered with entacapone)

DA-LEDD (mg) = pramipexole × 100 + piribedil × 1 + rotigotine × 30 + ropinirole × 20

Total LEDD (mg) = levodopa-LEDD + DA-LEDD + selegiline × 10 + rasagiline × 100 + amantadine × 1

#### 2.2.2 Scale assessments

All enrolled patients underwent the following clinical scale assessments:

(1) Motor symptoms and staging

Motor symptom severity was assessed using Part III of the Unified Parkinson's Disease Rating Scale (UPDRS-III). Disease severity staging was assessed using the revised Hoehn-Yahr (H–Y) staging scale (Goetz et al., 2008).

(2) Motor complications

Wearing-off phenomena and dyskinesia were separately assessed according to the relevant items in Part IV of the UPDRS. Wearing-off phenomena were classified as positive if present; dyskinesia was classified as positive if present. Freezing of gait (FOG) was assessed using item 11 of UPDRS Part III, and was classified as positive if present (Goetz et al., 2008).

(3) Non-motor symptoms

Cognitive function: Global cognitive function was assessed using the Mini-Mental State Examination (MMSE) and the Montreal Cognitive Assessment (MoCA).

Orthostatic hypotension: Orthostatic hypotension was assessed using supine-to-standing blood pressure measurements. Blood pressure was measured after supine rest, immediately upon standing, and at 1 and 3 min after standing. Orthostatic hypotension was defined as a drop in systolic blood pressure of ≥20 mmHg or a drop in diastolic blood pressure of ≥10 mmHg. For patients with concomitant supine hypertension, a drop in systolic blood pressure of ≥30 mmHg was used as the criterion (Freeman et al., 2011).

Constipation: Constipation was assessed according to the Rome IV diagnostic criteria for functional constipation. Participants were required to meet at least two of the following criteria, with symptoms persisting for ≥6 months, and irritable bowel syndrome being excluded: (1) straining during >25% of bowel movements; (2) lumpy or hard stools (Bristol Stool Form Scale types 1–2) for >25% of bowel movements; (3) sensation of incomplete evacuation for >25% of bowel movements; (4) sensation of anorectal obstruction for >25% of bowel movements; (5) manual maneuvers to facilitate >25% of bowel movements; (6) fewer than three spontaneous bowel movements per week (Warnecke et al., 2022).

REM sleep behavior disorder (RBD): RBD was assessed using the RBDQ-HK questionnaire (positivity was defined as a total score ≥18 and/or a factor 2 score ≥7), which is a screening tool rather than a confirmatory diagnostic method such as polysomnography (PSG).

Activities of Daily Living: basic activities of daily living (BADL) were assessed using the Basic Activities of Daily Living scale to evaluate fundamental self-care abilities (eating, dressing, transfer, toileting). Instrumental activities of daily living (IADL) were assessed using the Instrumental Activities of Daily Living scale to evaluate more complex abilities (shopping, meal preparation, medication management, financial management).

#### 2.2.3 fNIRS assessment

##### 2.2.3.1 fNIRS data acquisition

A 48-channel fNIRS system (Nirscan-6,000A, DanYang HuiChuang Medical Equipment Co., Ltd., China) was used in this study. Signals reflecting brain functional activity were acquired at a sampling rate of 11 Hz, using two wavelengths of 730 nm and 850 nm. According to the international 10–20 electrode placement system, 32 probes (16 emitters and 16 detectors) were placed over the frontal, parietal, and motor-related cortices, forming 48 channels with an inter-probe distance of 30 mm. Probe positions were registered to the standard MNI template via virtual co-registration based on anatomical landmarks. Data acquisition was performed in a quiet, dimly lit environment. To ensure that participants could perform the grasping task optimally, all measurements were conducted during the medication “ON” state, i.e., 1–2 h after the intake of anti-Parkinsonian medication. No short-channel correction was applied (30 mm source-detector distance > typical short-channel threshold); instead, scalp blood flow influence was minimized through the preprocessing and QC procedures described below.

##### 2.2.3.2 fNIRS task paradigm

Before measurement, all participants were asked to remain still and to confirm the testing procedure with the researcher. During testing, participants were required to refrain from speaking and to maintain a relaxed seated position. Participants performed the hand grasping task sequentially with the right and left hands. During the task, participants were instructed to keep the ipsilateral upper arm, forearm, and other limbs relaxed and still, while performing hand grasping movements at a frequency of 1 Hz.

A block design was adopted. Each task block lasted 45 s, consisting of a 25-s rest phase (instruction: “rest”) followed by a 20-s motor phase (instruction: “grasp”), during which participants performed hand grasping movements following a video demonstration. The task was repeated five times. Before the first task block, a 10-s rest phase was set as the baseline. The entire testing procedure lasted 245 s (see Figure 1).

**Figure 1:**
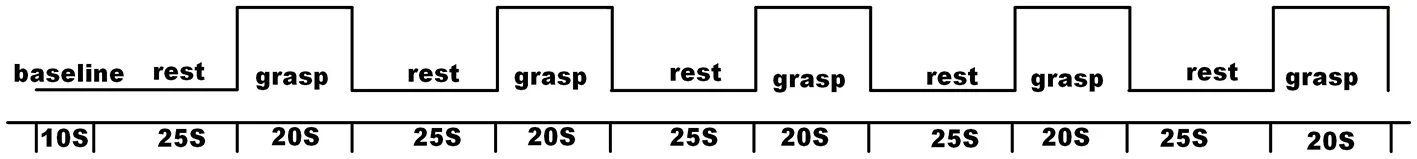
Schematic diagram of the fNIRS experimental paradigm for the hand grasping task. Each task block consisted of a 25-s rest phase followed by a 20-s motor phase (hand grasping at 1 Hz), repeated five times. The experiment began with a 10-s baseline rest phase.

##### 2.2.3.3 fNIRS data preprocessing

fNIRS data were preprocessed using the preprocessing module of NirSpark software. The preprocessing procedure consisted of the following steps:

**Motion artifact correction:** in the Motion Correction module, the “Auto” mode was selected, with the standard deviation threshold (SD_thr) set to 6 and the amplitude threshold (amp_thr) set to 0.5. Motion artifacts were removed using a combination of moving standard deviation and cubic spline interpolation. To further ensure data quality, trials exhibiting signal characteristics consistent with excessive head motion (relative displacement >0.2 mm) were excluded, following quality control criteria originally established in fMRI research and subsequently adopted in fNIRS studies (Power et al., 2013)

**Filtering:** in the Filter module, a bandpass filter of 0.01–0.2 Hz was applied to the data to remove low-frequency drift caused by spontaneous physiological and environmental changes, as well as high-frequency noise and physiological fluctuations (e.g., heartbeat and respiration) (Pinti et al., 2018).

**Concentration conversion:** in the Hemo module, the differential path factor (DPF) was set to 6, 6, 6. The optical density signals were converted into concentration changes of oxygenated hemoglobin (HbO_2_) and deoxygenated hemoglobin (HbR) according to the modified Lambert-Beer law.

##### 2.2.3.4 Region of interest definition

The regions of interest (ROIs) defined in this study included the primary somatosensory cortex (S1), primary motor cortex (M1), superior anterior cingulate cortex (SAC), premotor cortex (PMC), frontal eye field (FEF), supplementary motor area (SMA), and dorsolateral prefrontal cortex (DLPFC). Channels were assigned to ROIs based on probabilistic registration of each channel's sensitivity profile to the MNI space, with ROI definitions derived from standard anatomical atlases (Brodmann areas) and anatomical landmarks. The channel numbers corresponding to each ROI are presented in Table 1, and the spatial distribution of the channels is illustrated in Figure 2.

**Table 1:** Channel numbers corresponding to each region of interest (ROI).

ROI	Channel numbers
L-S1	11, 14, 16, 17, 29
R-S1	41, 44, 42, 43, 31
L-M1	3, 10, 13, 16, 25, 28
R-M1	48, 40, 39, 42, 27, 30
L-SAC	15, 17, 29
R-SAC	45, 43, 31
L-PMC	2, 6, 7, 9, 10, 12, 13
R-PMC	47, 36, 37, 35, 40, 38, 39
L-FEF	5, 8, 18
R-FEF	32, 34, 19
L-SMA	20, 21, 24, 25
R-SMA	22, 23, 26, 27
L-DLPFC	1, 4, 6
R-DLPFC	46, 33, 36

**Figure 2:**
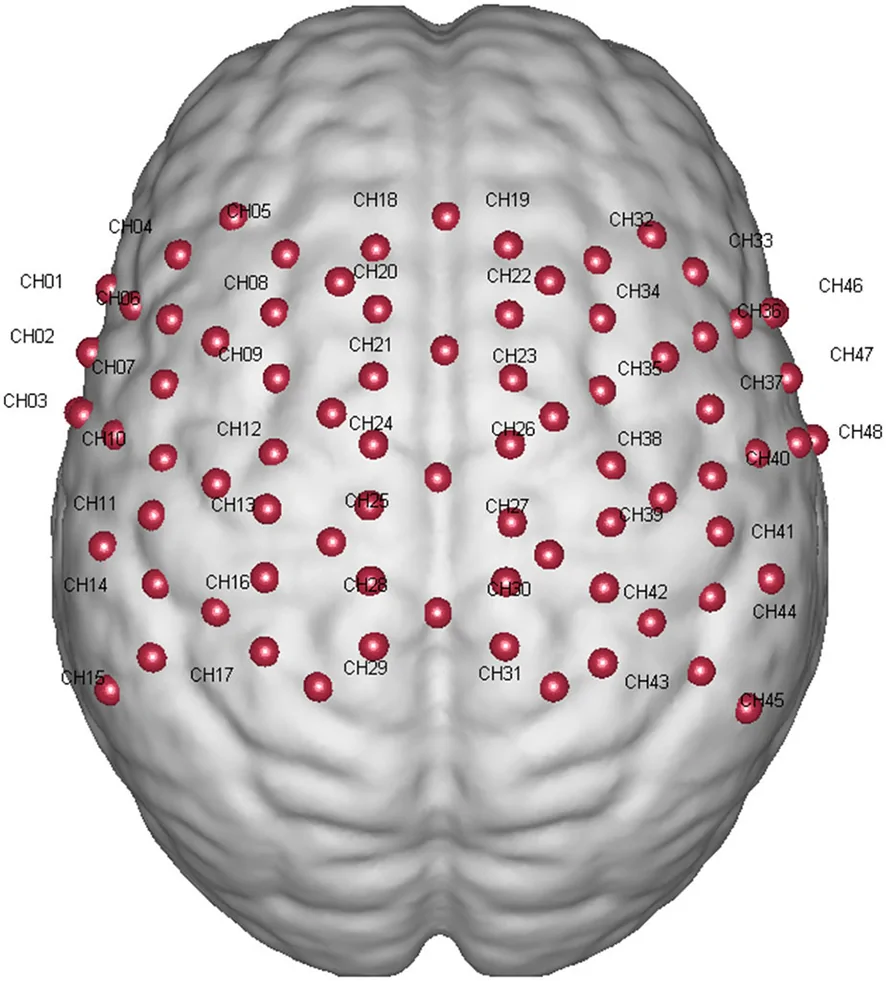
Spatial distribution of fNIRS channels: this figure was exported from the fNIRS device software, showing the spatial projection positions of the 48 channels (CH01–CH48) on the scalp surface. The numbers in the figure indicate channel numbers. The top of the figure corresponds to the nasion direction; the left side represents the left hemisphere **(L)** and the right side represents the right hemisphere **(R)**. The channel combinations corresponding to each region of interest (ROI) are detailed in Table 1.

##### 2.2.3.5 Data quality control and feature extraction

After preprocessing, data quality control (QC) was performed using Python. Bad trials, abnormal regions of interest (ROIs), and unqualified participants were sequentially excluded, ultimately yielding the QC-passed participant data (Subject_mean_qc).

A robust z-score based on the median and median absolute deviation (MAD) was used to assess the quality of each trial-ROI combination. The evaluation metrics included: baseline standard deviation, standard deviation of high-frequency fluctuations, signal amplitude range, absolute value of signal drift slope, and absolute peak value during the task period. A trial-ROI combination was labeled as “bad” if any robust z-score exceeded 3.5, or if the absolute peak value during the task period exceeded 0.25 mM·mm (this threshold was used to exclude large-amplitude noise interference that might occur during the task period).

The QC criteria were as follows: If the proportion of bad trials for a given ROI in a participant was ≥40%, that ROI-participant combination was marked as unreliable. If the proportion of unreliable ROIs in a participant was ≥40%, that participant was entirely excluded from subsequent analyses. Applying these criteria, 108 ROI–subject combinations (12.6%) were identified as bad and excluded during the right-hand task, and 98 ROI–subject combinations (10.9%) during the left-hand task; across both tasks, a total of 902 trials (10.7%) were excluded, and 13 participants failed QC in at least one of the two tasks. After taking the union of participants with valid data for at least one hand task, 59 participants were included in the final analysis. After QC, only valid trials were retained for calculating the mean HbO_2_ concentration; ROI-participant combinations marked as unreliable were retained as missing values (NaN) in subsequent analyses.

To visually demonstrate the task-related hemodynamic response characteristics of the QC-passed data, a representative participant who passed all QC criteria was selected, and the hemodynamic response curves of each brain region during the right-hand grasping task were plotted (Figure 3). The results showed that in the sensorimotor cortices (S1, M1, SAC), HbO_2_ increased with a corresponding decrease in HbR after task onset, followed by a peak and then a return to baseline, exhibiting a regular pattern consistent with normal cerebral hemodynamic responses during hand motor tasks.

**Figure 3:**
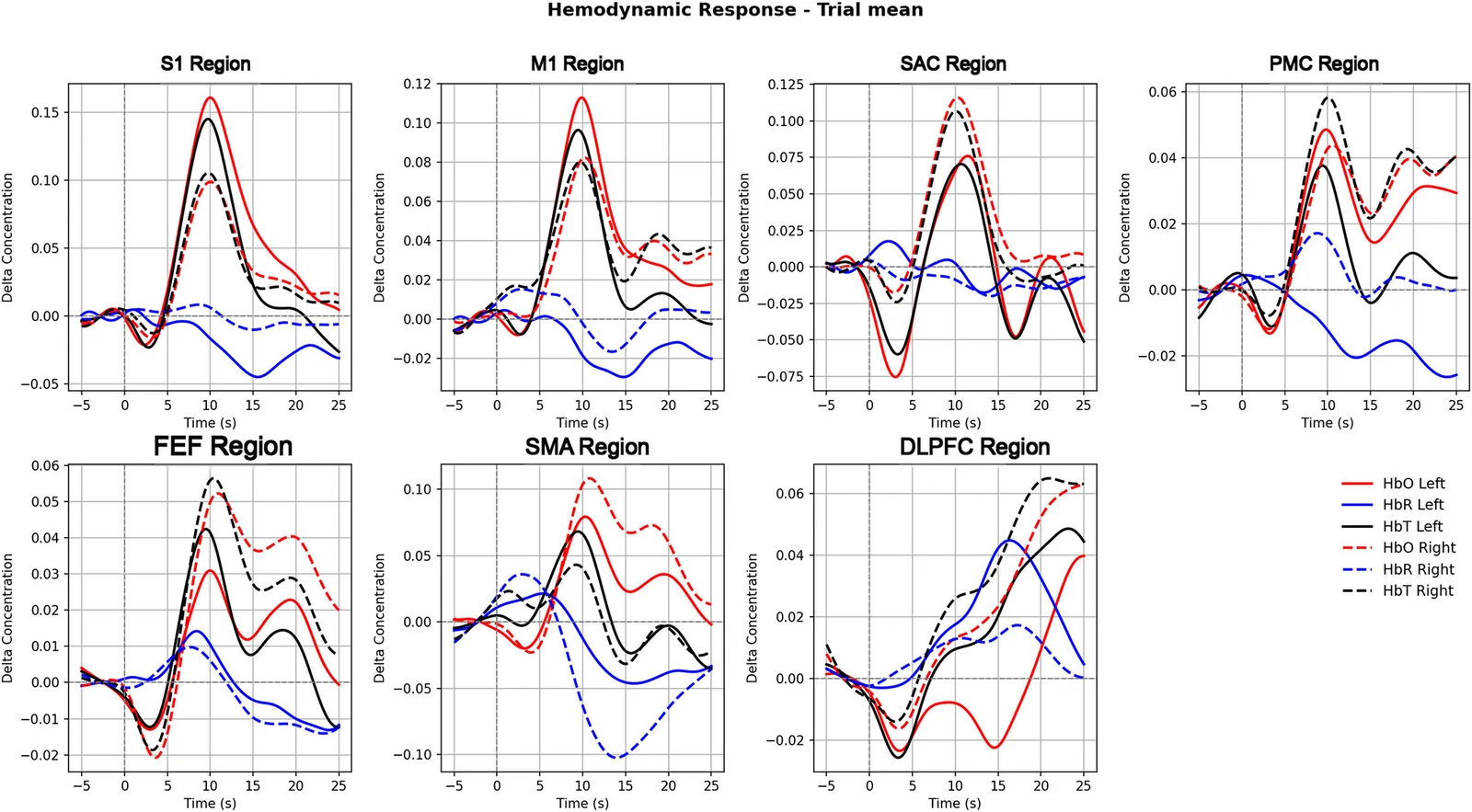
Hemodynamic response curves during the right-hand grasping task in a QC-passed participant: this figure shows the mean hemodynamic response curves of different brain regions of interest (S1, M1, SAC, PMC, FEF, SMA, DLPFC) in a representative participant who passed all quality control criteria while performing the right-hand grasping task. Red solid/dashed lines represent left/right oxygenated hemoglobin (HbO_2_) concentration changes, blue solid/dashed lines represent left/right deoxygenated hemoglobin (HbR) concentration changes, and black solid/dashed lines represent left/right total hemoglobin (HbT) concentration changes. The X-axis represents time (seconds, with 0 s as the task onset), and the Y-axis represents the change in hemoglobin concentration (ΔConcentration). The curves show that in the sensorimotor cortices (S1, M1, SAC), HbO_2_ exhibited a typical “rise-peak-return” activation pattern after task onset, accompanied by a synchronous decrease in HbR. These findings confirm that the task paradigm used in this study effectively elicits brain activation, and the overall data quality meets the requirements for subsequent analyses.

Feature extraction was performed using a time window of 0–25 s after baseline. Because the hemodynamic response typically exhibits a lag (approximately 4–6 s to peak) and requires time to return to baseline, the time window was extended to 25 s to fully capture the rising and falling phases of activation. Both temporal-domain features and advanced features were extracted.

##### 2.2.3.5.1 Temporal-domain features

**Peak:** the maximum HbO_2_ concentration during the task period, reflecting the intensity of brain activation.

**Time to peak (TTP):** the time (in seconds) from task onset to the peak value, reflecting the response speed of brain activation.

**Mean:** the average HbO_2_ concentration across the entire task period.


$$\begin{array}{l} mean=\frac{1}{n}\overset{n}{\underset{i=1}{\sum }}x_{i} \end{array}$$

**Standard deviation (SD):** the degree of fluctuation in HbO_2_ concentration during the task period, reflecting the variability of activation.


$$\begin{array}{l} std=\sqrt{\frac{1}{n-1}\overset{n}{\underset{i=1}{\sum }}{\left(x_{i}-\bar{x}\right)}^{2}} \end{array}$$

$$\begin{array}{l} \bar{x}=mean \end{array}$$

**Area under the curve (AUC):** the integral of HbO_2_ concentration over time, reflecting the total amount of activation.


$$\begin{array}{l} AUC=\int_{t_{1}}^{t_{2}}HbO\left(t\right)dt \end{array}$$

##### 2.2.3.5.2 Advanced features

**Slope:** the rising rate from task onset to the peak, reflecting the “initiation speed” of brain activation. The calculation formula is:


$$\begin{array}{l} slope=\frac{HbO_{peak}-HbO_{baseline}}{t_{peak}-t_{start}} \end{array}$$

**Full width at half maximum (FWHM):** the duration (in seconds) at half of the peak height, reflecting the temporal persistence of activation.

**Peak count:** the number of significant peaks during the task period.

### 2.3 Statistical analysis

**Clinical data statistical analysis:** clinical data were analyzed using SPSS version 26.0. Continuous variables were described as mean ± standard deviation, and categorical variables were described as counts (percentages) [*n* (%)].

**fNIRS data analysis:** fNIRS data preprocessing was performed using NirSpark software, and feature extraction and visualization were implemented using Python software.

**Missing value imputation and regression analysis:** missing values in clinical variables and fNIRS features were handled using multiple imputation by chained equations (MICE) with 5 imputations. The imputation model included all analytical variables in this study, and the final results were pooled according to Rubin's rules.

Multiple linear regression models were used to explore the associations between clinical indices and brain activation levels in PD patients. Each clinical index was entered as an independent variable, with age, sex, and disease duration as covariates, and regressed against each of the 112 fNIRS brain activation parameters derived from 14 regions of interest under the left- and right-hand task conditions (i.e., 112 separate regression models). All categorical independent variables were converted into dummy variables before being entered into the models. Multiple comparisons were corrected within each clinical variable using the Benjamini-Hochberg false discovery rate (FDR) method. A corrected *p*-value (pFDR) < 0.05 was considered statistically significant. The sign (positive or negative) of the regression coefficient was used to determine the direction of the effect of clinical variables on cerebral oxygenation activation.

## 3 Results

### 3.1 Clinical characteristics

A total of 59 PD patients were included in the final clinical data analysis, consisting of 32 males (54.2%) and 27 females (45.8%). The patients' age ranged from 51 to 83 years, with a mean age of 66.20 ± 7.76 years. Years of education ranged from 0 to 16 years (mean: 10.76 ± 4.34 years). Disease duration ranged from 0.50 to 17.00 years (mean: 5.45 ± 3.70 years). The Hoehn-Yahr (H–Y) stage ranged from 1.0 to 4.0 (mean: 2.14 ± 0.73), and the UPDRS-III score ranged from 12 to 98 (mean: 37.66 ± 16.04).

Regarding clinical characteristics, 17 patients (28.8%) presented with freezing of gait (FOG), 38 patients (64.4%) had constipation, and 17 patients (28.8%) had REM sleep behavior disorder (RBD). Detailed clinical data are presented in Table 2.

**Table 2:** Clinical characteristics of Parkinson's disease patients.

Variable	PD group (*n* = 59)	Range
Age (years)	66.20 ± 7.76	51–83
Male [*n* (%)]	32 (54.2%)	-
Education (years)	10.76 ± 4.34	0–16
Disease duration (years)	5.45 ± 3.70	0.50–17.00
H–Y stage	2.14 ± 0.73	1.0–4.0
UPDRS–III score	37.66 ± 16.04	12–98
Right–sided onset [*n* (%)]	33 (55.9%)	-
Tremor onset [*n* (%)]	42 (71.2%)	-
Rigidity onset [*n* (%)]	15 (25.4%)	-
Bradykinesia onset [*n* (%)]	2 (3.4%)	-
Wearing–off phenomenon [*n* (%)]	33 (55.9%)	-
Dyskinesia [*n* (%)]	11 (18.6%)	-
Freezing of gait (FOG) [*n* (%)]	17 (28.8%)	-
MMSE score	25.04 ± 4.52	10–30
MoCA score	20.66 ± 6.00	7–30
BADL score	7.84 ± 3.30	6–20
IADL score	12.48 ± 7.07	7–30
Orthostatic hypotension (OH) [*n* (%)]	16 (27.1%)	-
Constipation [*n* (%)]	38 (64.4%)	-
RBD [*n* (%)]	17 (28.8%)	-

Continuous variables were described as mean ± standard deviation (SD), and categorical variables were described as counts (percentages) [*n* (%)].

### 3.2 Associations between clinical variables and brain activation

After handling missing values using multiple imputation by chained equations (MICE), multiple linear regression analyses were performed with age, sex, and disease duration as covariates to examine the associations between clinical variables (constipation, RBD, FOG, dyskinesia, wearing-off phenomenon, side of onset, UPDRS-III, H-Y stage, MMSE, MoCA, LEDDmg, DALEDDmg, tLEDDmg) and 112 brain activation parameters (14 regions of interest × 8 features). The false discovery rate (FDR) method was used for multiple comparison correction.

#### 3.2.1 Constipation and brain activation

During the left-hand grasping task, constipation was significantly positively correlated with activation parameters in multiple prefrontal and motor-related brain regions (FDR-corrected *p* < 0.05). As shown in Figure 4, constipation-related significant channels (red dots) were mainly distributed in the bilateral dorsolateral prefrontal cortex, left frontal eye field, and left premotor cortex. Quantitative analysis of the associations further revealed that constipation was significantly positively correlated with 13 activation indices in the above-mentioned brain regions: the left dorsolateral prefrontal cortex (L_DLPFC) involved five indices [peak, mean, standard deviation (SD), area under the curve (AUC), slope]; the left frontal eye field (L_FEF) involved three indices (peak, mean, SD); the left premotor cortex (L_PMC) involved two indices (peak, SD); and the right dorsolateral prefrontal cortex (R_DLPFC) involved three indices (peak, mean, SD). All significant associations had positive regression coefficients (range: 0.0033–0.8538), as detailed in Table 3 and Figure 5.

**Figure 4:**
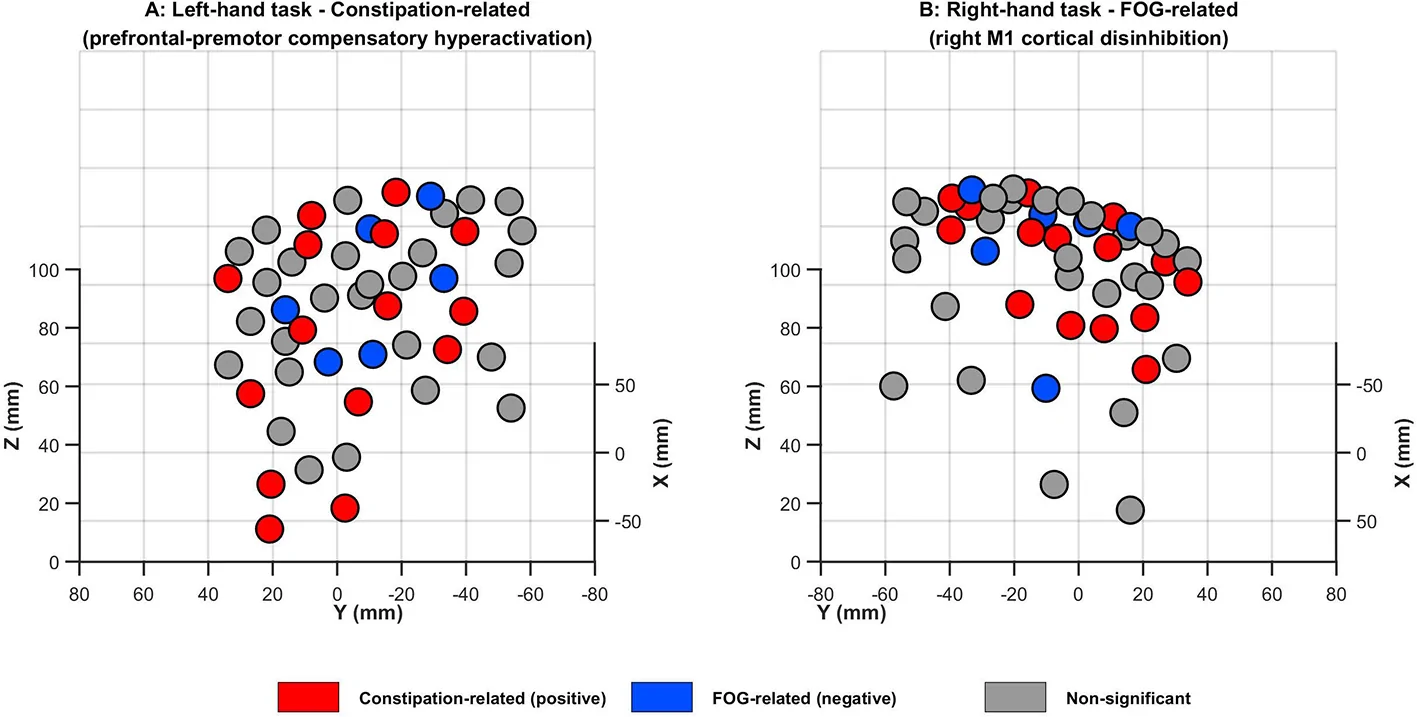
Phenotype-specific cortical activation patterns during hand grasping in PD patients. Each dot represents one fNIRS measurement channel (48 channels in total). **(A)** Left-hand task: red dots indicate channels showing significant positive associations between constipation and activation parameters of the prefrontal-premotor network (pFDR < 0.05). **(B)**Right-hand task: the blue dot indicates the channel showing a significant negative association between freezing of gait (FOG) and the full width at half maximum (FWHM) of the right primary motor cortex (M1) (pFDR =4 0.010). Gray dots indicate channels with no significant associations. L, left; R, right.

**Table 3:** Significant associations between clinical variables and brain activation parameters (FDR–corrected).

Task	Variable	BrainRegion	Indicator	β	95% CI	pFDR
Left	Constipation	L_DLPFC	peak	0.0461	[0.024,0.068]	0.019
Left	Constipation	L_FEF	peak	0.0293	[0.014,0.045]	0.020
Left	Constipation	L_PMC	peak	0.0347	[0.012,0.057]	0.038
Left	Constipation	R_DLPFC	peak	0.0279	[0.013,0.043]	0.020
Left	Constipation	L_DLPFC	mean	0.0341	[0.011,0.058]	0.031
Left	Constipation	L_FEF	mean	0.0235	[0.008,0.039]	0.037
Left	Constipation	R_DLPFC	mean	0.0254	[0.010,0.041]	0.020
Left	Constipation	L_DLPFC	std	0.0122	[0.006,0.019]	0.020
Left	Constipation	L_PMC	std	0.0102	[0.003,0.017]	0.048
Left	Constipation	L_DLPFC	auc	0.8538	[0.254,1.454]	0.031
Left	Constipation	L_FEF	auc	0.5910	[0.191,0.991]	0.037
Left	Constipation	R_DLPFC	auc	0.6445	[0.242,1.047]	0.020
Left	Constipation	L_DLPFC	slope	0.0033	[0.001,0.006]	0.046
Right	FOG	R_M1	fwhm	−8.5202	[−12.98,−4.06]	0.010

Abbreviations in the table are defined in the main text.

**Figure 5:**
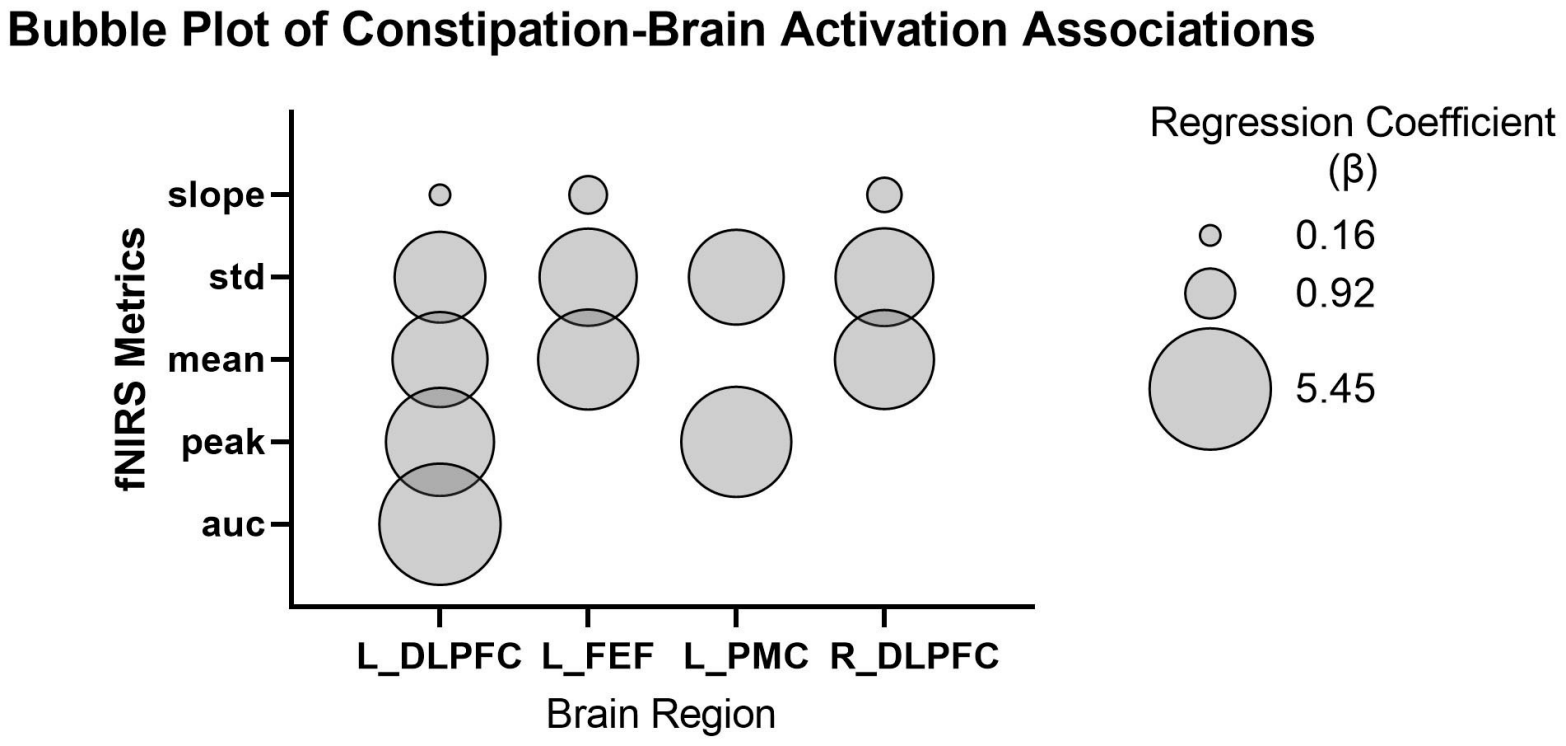
Significant associations between constipation and brain activation indices during the left-hand task (FDR-corrected *p* < 0.05): bubble size represents the natural logarithm-transformed regression coefficient (lnβ), with larger bubbles indicating stronger associations. All associations were positive. Raw β values are presented in Table 3. The x-axis shows fNIRS activation indices, and the y-axis shows brain regions.

During the right-hand grasping task, no significant associations were found between constipation and any brain activation indices (FDR-corrected *p* > 0.05).

#### 3.2.2 Freezing of gait and brain activation

During the left-hand grasping task, no significant associations were found between freezing of gait (FOG) and any brain activation parameters (FDR-corrected *p* > 0.05).

During the right-hand grasping task, FOG was significantly negatively correlated with the full width at half maximum (FWHM) of the right primary motor cortex(*R*_*M*_1) (β = −8.5202, 95% CI: −12.98 to −4.06, FDR-corrected *p* = 0.010) (see Table 3, Figure 4).

#### 3.2.3 RBD and brain activation

In uncorrected analyses, RBD positivity showed nominal associations with several brain activation parameters (*p* < 0.05, uncorrected), as detailed below (see Table 4).

**Table 4:** Associations between RBD and brain activation parameters (uncorrected *p* < 0.05).

Task	Variable	BrainRegion	Indicator	β	95% CI	*p* (uncorrected)
Left	RBD	L_FEF	Mean	0.018	[0.003, 0.034]	0.023
Left	RBD	L_FEF	Auc	0.458	[0.063, 0.853]	0.024
Left	RBD	R_PMC	Mean	0.022	[0.004, 0.041]	0.019
Left	RBD	R_PMC	peak	0.025	[0.003, 0.048]	0.030
Left	RBD	R_PMC	auc	0.556	[0.091, 1.021]	0.020
Left	RBD	R_M1	mean	0.021	[0.001, 0.041]	0.041
Left	RBD	R_M1	peak	0.025	[0.000, 0.050]	0.048
Left	RBD	R_M1	auc	0.514	[0.016, 1.013]	0.044
Left	RBD	R_M1	time_to_peak	5.614	[1.563, 9.664]	0.008
Left	RBD	L_PMC	time_to_peak	5.245	[1.613, 8.878]	0.005
Left	RBD	L_FEF	peak	0.019	[0.001, 0.037]	0.038
Left	RBD	L_FEF	fwhm	3.816	[0.214, 7.418]	0.038
Left	RBD	R_AFL	slope	0.002	[0.000, 0.004]	0.026
Left	RBD	R_FEF	slope	0.002	[0.000, 0.004]	0.042
Right	RBD	L_S1	time_to_peak	5.428	[1.513, 9.343]	0.008
Right	RBD	R_S1	time_to_peak	4.369	[0.601, 8.137]	0.024
Right	RBD	L_M1	time_to_peak	3.532	[0.159, 6.905]	0.041
Right	RBD	L_PMC	time_to_peak	3.910	[0.009, 7.811]	0.049
Right	RBD	R_SAC	time_to_peak	3.598	[0.119, 7.077]	0.043
Right	RBD	L_FEF	Std	0.008	[0.001, 0.014]	0.018

Abbreviations in the table are defined in the main text.

Left-hand grasping task: RBD positivity was associated with increased mean activation (β = 0.018, 95% CI: 0.003–0.034, *p* = 0.023) and area under the curve (AUC) (β = 0.458, 95% CI: 0.063–0.853, *p* = 0.024) in the left frontal eye field (FEF); increased AUC in the right primary motor cortex (M1) (β = 0.514, 95% CI: 0.016–1.013, *p* = 0.044); and increased peak activation in the right premotor cortex (PMC) (β = 0.025, 95% CI: 0.003–0.048, *p* = 0.030). In addition, RBD positivity was associated with prolonged time to peak and increased Full Width at Half Maximum (FWHM) in multiple bilateral brain regions.

Right-hand grasping task: RBD positivity was associated with prolonged time to peak in the left primary somatosensory cortex (S1) (β = 5.428, 95% CI: 1.513–9.343, *p* = 0.008), right S1 (β = 4.369, 95% CI: 0.601–8.137, *p* = 0.024), and right superior anterior cingulate cortex (SAC) (β = 3.598, 95% CI: 0.119–7.077, *p* = 0.043); and with increased standard deviation (SD) in the left frontal eye field (FEF) (β = 0.008, 95% CI: 0.001–0.014, *p* = 0.018).

None of the above associations survived FDR correction for multiple comparisons (pFDR > 0.05), highlighting the limited sensitivity of superficial fNIRS signals to the deep subcortical pathology of RBD.

#### 3.2.4 Other clinical variables

No significant associations were found between any of the following clinical variables and any brain activation parameters after FDR correction (all corrected *p* > 0.05): dyskinesia, wearing-off phenomenon, motor severity (UPDRS-III, H–Y stage), cognitive function (MMSE, MoCA), and medication dosage (LEDD, DA-LEDD, total LEDD).

## 4 Discussion

The main findings of this study are as follows: (1) During the non-dominant hand grasping task, constipation was significantly associated with cortical hyperactivation in the bilateral dorsolateral prefrontal cortex and premotor cortex, manifested as concurrent increases in peak, mean, area under the curve, standard deviation, and slope. (2) During the dominant hand grasping task, freezing of gait (FOG) was significantly associated with a narrowed full width at half maximum (FWHM) in the right primary motor cortex (M1), suggesting altered temporal dynamics of the hemodynamic response. (3) After rigorous multiple comparison correction, neither REM sleep behavior disorder (RBD) nor other conventional clinical variables (motor severity, cognitive function, levodopa dosage, etc.,) showed independent significant associations with cortical activation parameters. These findings reveal phenotype-specific cortical modulation patterns for constipation and FOG in PD, providing objective neuroimaging evidence for clinical subtyping and generating testable hypotheses for individualized rTMS targeting.

### 4.1 Constipation and cortical activation of the dorsolateral prefrontal-premotor cortex during the non-dominant hand task

Previous resting-state fMRI studies have demonstrated significant abnormalities in viscero-emotional brain regions (e.g., insula, orbitofrontal cortex) in PD patients with constipation, indirectly suggesting a functional link between gut autonomic imbalance and central circuit remodeling (Zheng et al., 2022; Su et al., 2024). However, task-based motor paradigms investigating the temporal dynamics of cortical activation associated with constipation remain lacking. In our study, PD patients with constipation exhibited significantly higher peak activation, mean activation, and area under the curve (AUC) in the bilateral dorsolateral prefrontal cortex (DLPFC) and premotor cortex (PMC) during the non-dominant hand grasping task compared to those without constipation. According to the Braak gut-brain axis hypothesis, gut-derived α-synuclein can retrogradely affect the central nervous system via the vagus nerve, progressively disrupting the functional integrity of the DLPFC-PMC circuit (Braak et al., 2003), thereby inducing compensatory cortical hyperactivation—a pattern consistent with the known characteristics of cortical functional remodeling in prodromal PD (van Nuenen et al., 2012). We also found that PD patients with constipation showed increased fluctuation amplitude (standard deviation) of HbO_2_ signals in the DLPFC and a significantly steeper activation slope during the task initiation phase, indicating both reduced stability and accelerated neural recruitment. Wolpe et al. noted that when fronto-striatal functional reserve is reduced, the brain increasingly relies on real-time sensory feedback rather than internal motor predictions, leading to elevated cortical signal variability (Wolpe et al., 2016)—a finding that aligns with our observation of increased standard deviation and steeper slope in the left DLPFC of constipated patients. Helmich et al. similarly reported reduced basal ganglia function accompanied by compensatory frontal activation in PD (Helmich et al., 2009). Notably, the constipation-related cortical abnormalities were observed exclusively during the non-dominant hand task. This finding aligns with the framework of hemispheric asymmetry in PD motor control: the dominant hemisphere is thought to possess greater neural reserve (Ham et al., 2015), whereas the non-dominant hemisphere has weaker cortical representation, rendering its motor network more vulnerable to basal ganglia dysfunction—consistent with evidence that the left (dominant) hemisphere exerts stronger ipsilateral control over upper-limb movements in right-handed individuals (Stewart et al., 2009). Recent reviews have further underscored the clinical significance of this lateralization, demonstrating its influence on both motor and non-motor symptom profiles (Steinbach et al., 2023). Under this framework, the higher regulatory load of the non-dominant hand task may more readily expose constipation-related functional deficits, as the compromised basal ganglia-thalamo-cortical loop is challenged beyond its compensatory capacity—supporting the view that motor asymmetry in PD extends to autonomic regulation, including gastrointestinal function. Collectively, our task-based findings complement previous resting-state evidence, providing new hemodynamic evidence for systemic central nervous system impairment in PD. The results further support an association between constipation and reduced functional reserve of the fronto-striatal network, as well as abnormal DLPFC-PMC circuit activation. For PD patients with comorbid constipation, the DLPFC may represent a candidate target for rTMS intervention to improve both motor and non-motor symptoms—a hypothesis warranting validation in future interventional trials.

### 4.2 fNIRS-derived reduced hemodynamic persistence in the right primary motor cortex during the dominant hand task in freezing of gait

From the perspective of cortical hemodynamic regulation, a reduction in full width at half maximum (FWHM) suggests reduced hemodynamic persistence, reflecting altered temporal dynamics of the cortical response. This abnormality further compresses the effective output duration of motor commands, ultimately resulting in reduced motor regulation efficiency (Vitorio et al., 2025) In this study, we found that patients with freezing of gait (FOG) exhibited a significantly reduced FWHM in the right primary motor cortex (M1), consistent with the above mechanism, suggesting that FOG-related hemodynamic persistence deficits shorten the effective activation duration of M1 and impair fine motor output regulation. Using transcranial magnetic stimulation (TMS), Sun et al. (2023) demonstrated significantly reduced short-interval intracortical inhibition (SICI) in patients with FOG, confirming functional abnormalities of cortical inhibitory interneurons in this population, which are closely associated with the progression of gait disturbances. According to the SCAN network theory, M1 serves as a core node for motor output; an imbalance in its excitatory-inhibitory equilibrium can lead to motor initiation delay, rhythm disturbances, and gait freezing, representing an important cortical pathological basis for FOG. These findings support our observation of altered hemodynamic persistence in M1 and highlight the value of multi-region assessments in research on motor disorders in Parkinson's disease. Recent work has reported aberrant M1-SMA connectivity in FOG patients during sensory-contextual walking paradigms, with exploratory stepwise regression linking this metric to in-task freezing episodes in the medication-OFF state (Chen et al., 2025). Our study further confirmed that the aforementioned cortical functional abnormalities exhibit dominant hand task specificity. The dominant hand motor control pattern is more automated and thus more sensitive to the stability of brain networks and the precision of neural signal output. When the functional reserve of the SCAN network is reduced, diminished hemodynamic persistence in M1 becomes more readily exposed (Cockx et al., 2024). Previous behavioral studies have also indicated that PD patients with comorbid FOG exhibit significant motor control impairments during upper-limb force regulation tasks, suggesting that the pathological damage of FOG is not limited to the lower limbs but also involves upper-limb motor control circuits (Aitken et al., 2024). In summary, FOG is not a lower-limb-specific disorder but a manifestation of widespread motor cortical dysfunction. M1, as the final common pathway for motor output, is a key node in this network. Our findings link FOG to shortened M1 hemodynamic activation duration. Although our upper-limb paradigm cannot directly capture gait-freezing, it offers a probe for M1 hemodynamic persistence deficits. Thus, right M1 rTMS may restore network balance, and FWHM may serve as a treatment biomarker—hypotheses warranting testing in future walking-task interventional studies.

### 4.3 Weak cortical effects of REM sleep behavior disorder and limitations of fNIRS detection

As a characteristic clinical marker of the body-first subtype of Parkinson‘s disease, RBD has significant value for disease subtyping and early identification. Unlike constipation and FOG—which show clear cortical activation—RBD pathology primarily involves deep brain regions (brainstem, thalamus), and its cortical activity features are weakly stable and poorly reproducible (Ellmore et al., 2013; Qin et al., 2025), making them difficult to detect with task-based fNIRS. In our study, analyses without multiple comparison correction revealed only weak and scattered hemodynamic trends in RBD-related sensorimotor regions; after FDR correction, no significant associations remained. This is consistent with most PD-fNIRS studies (Chen Thomsen et al., 2024; Hui et al., 2024). However, given the small RBD subgroup (*n* = 17), the possibility of subtle cortical effects being missed due to limited statistical power cannot be excluded. These findings should therefore be interpreted cautiously, and larger studies are needed to further evaluate the cortical correlates of RBD.

### 4.4 Interpretation of negative findings for other clinical variables

Previous studies on clinical variables—including side of onset, motor severity, dyskinesia, wearing-off, cognition, and levodopa dosage—have reported inconsistent associations with brain activation (Tahmasian et al., 2017; Xing et al., 2020; Chen Thomsen et al., 2024). In our study, after adjusting for age, sex, and disease duration, none of these variables showed independent or stable associations with cortical activation, suggesting that traditional motor phenotypes and pharmacological measures alone cannot fully explain the individual heterogeneity in cortical activation in PD. This negative finding may be explained by multicollinearity among clinical indices and the SCAN network framework. Most motor- and medication-related variables are highly collinear with disease duration and severity. For instance, levodopa dosage increases with disease duration, and its apparent effect largely depends on confounding variables; after covariate adjustment, its independent effect nearly disappears. PD is a systemic neurodegenerative disorder affecting multiple circuits, and superficial cortical activation is not directly dominated by any single motor index or medication dose. Collectively, our findings—consistent with Hui et al. (Hui et al., 2024)—suggest that individual differences in cortical activation may be more closely associated with non-motor symptom-related network dysfunction than with conventional motor phenotypes or levodopa dose. We acknowledge, however, that our regression models included only a limited set of covariates, which may have introduced residual confounding; future larger-scale studies with more comprehensive adjustment are needed to confirm these findings. Despite their biomarker potential, the clinical translation of fNIRS-derived metrics faces implementation challenges, including integration into workflows and acceptance among clinicians and patients. User acceptance and human-AI collaboration are critical for successful translation (Du et al., 2026a), and future research should combine methodological refinement with implementation science to facilitate the translation of objective neuroimaging biomarkers into personalized PD management.

Several limitations should be acknowledged. First, fNIRS detects only superficial cortical oxygenation signals and cannot probe deep brain structures (e.g., brainstem, basal ganglia, thalamus). Channel coverage excluded prefrontal subregions such as the ventromedial prefrontal cortex, orbitofrontal cortex, and frontal pole; thus, findings cannot be generalized to deep structures or the entire prefrontal cortex. Second, the cross-sectional design precludes causal inferences; only associations can be identified, and longitudinal studies are needed to establish temporality. Third, the hand grasping task limits generalizability to other domains (e.g., gait, speech, cognition) or resting-state conditions. Fourth, RBD was assessed via questionnaire (RBDQ-HK) rather than PSG; findings represent screening-defined positivity, not clinically confirmed RBD, which may have introduced classification bias. Fifth, although FDR correction was applied to 112 parameters, bootstrap resampling (1,000 iterations) and leave-one-out cross-validation confirmed the robustness of the 14 core associations (all retained their original direction; bootstrap 95% CIs did not cross zero). Additionally, adjusting for LEDD as an extra covariate left all 14 associations significant (100% retention), indicating that medication load does not substantially confound the results. Sixth, this single-center study with a homogeneous sample lacks external validation; multicenter studies are needed. Seventh, non-motor symptoms were treated as binary variables, precluding dose-response analyses. Eighth, FOG was assessed using binary UPDRS-III item 11, which does not capture severity; dedicated scales (e.g., FOG-Q) (Giladi et al., 2000) should be considered in future studies. Ninth, the absence of healthy controls and symptom-negative PD subgroups limits interpretation of whether observed hyperactivation is PD-specific, symptom-specific, or task-related. Additionally, the small FOG and RBD subgroups (*n* = 17 each) limited statistical power. A post-hoc sensitivity power analysis was performed: with 59 participants, the study was only powered to detect a Cohen's f^2^ of 0.38 after Bonferroni correction for 112 comparisons; for dichotomous variables with subgroup sizes of 17, the minimum detectable effect was *d* = 1.32. These results should be considered exploratory; null findings—particularly for RBD—may reflect Type II error rather than true absence of association. Future studies should expand sample sizes, optimize fNIRS channel placement, integrate multimodal analyses, include appropriate control groups, and conduct prospective multicenter designs to validate and extend these findings.

## 5 Conclusion

Using task-based fNIRS cerebral oxygenation metrics, this study investigated cortical activation characteristics and their independent influencing factors during hand grasping movements in patients with Parkinson's Disease (PD). The main conclusions are as follows:

1. PD patients with constipation exhibited widespread cortical hyperactivation, abnormal fluctuations in oxygenation signals, and accelerated neural recruitment during the task initiation phase while performing the non-dominant hand grasping task, suggesting progressive depletion of motor-related cortical functional reserve. These findings indicate that constipation may characterize the depletion state of the DLPFC-PMC network in PD. Within the body-first subtype framework, compared with RBD, constipation may serve as a more appropriate specific peripheral marker for assessing superficial cortical reserve impairment using fNIRS.
2. PD patients with Freezing of Gait (FOG) showed a significant narrowing of the Full Width at Half Maximum (FWHM) of signals in the right primary motor cortex (M1) and a marked shortening of the hemodynamic activation duration during the dominant hand grasping task. This pattern suggests reduced hemodynamic persistence and altered temporal dynamics of the cortical response, ultimately impairing motor output regulation. Therefore, the FWHM of the right M1 may serve as a neuroimaging biomarker for quantitatively assessing FOG-related cortical dysfunction and provides a potential candidate brain region that warrants further investigation for individualized rTMS targeting.
3. After rigorous multiple comparison correction, no significant differences in brain activity were observed between RBD-positive and RBD-negative PD patients in the present sample. However, given the limited statistical power, these negative findings should be interpreted with caution and do not exclude the possibility of subtle RBD-related cortical effects.
4. Conventional clinical variables, including motor symptom severity, dyskinesia, cognitive level, and levodopa dosage, did not exhibit independent or stable regulatory effects on cortical activation. These findings suggest that the aforementioned variables alone may not fully account for the individual heterogeneity in cortical activation in PD and are consistent with the hypothesis that the core pathological changes in PD may involve systemic SCAN network dysfunction rather than being confined to localized organic lesions of motor nuclei.

## Data Availability

The raw data supporting the conclusions of this article will be made available by the authors, without undue reservation.
